# Supplementary material for: Electromagnetic scattering laws in Weyl systems
Source: Nat Commun. 2017 Nov 9;8:1388. doi: 10.1038/s41467-017-01533-0 (PMC5680227; doi:10.1038/s41467-017-01533-0)
Supplement: Supplementary file 1 — Supplementary Information [file 41467_2017_1533_MOESM1_ESM.pdf]

Supplementary Note 1: Conservation law of resonant scattering

As we discussed in the main text, the conservation law of resonant scattering applies to both classical and quantum resonances in any continuum with a well-defined dispersion relation. Here in this section, we will derive the conservation law using quantum electrodynamics.

To simplify the discussion, we consider a non-degenerate resonance embedded in a medium. The resonance frequency of the resonance is ω_0. We also incorporate the Lamb shift into the resonance frequency to simplify the discussion[1]. The dispersion relation of the medium is defined by $\omega(\mathbf{k})$, where $\mathbf{k}$ is the wave vector. The interaction between the resonance and the propagating waves in the medium is described by the interaction Hamiltonian H_{I}. The exact expression of H_{I} is not required to derive the conservation law as we will demonstrate below.

We consider the incident photon is in an eigenmode of the medium with a momentum of $\mathbf{k}_{\mathrm{i}}$. The resonance is initially in its ground state $|\mathrm{g}\rangle$. The incident photon is scattered by the resonance to all the eigenmodes of the medium. For a final state with a momentum of $\mathbf{k}_{\mathrm{f}}$, the transition probability is given by[2]

$$\mathfrak{I}_{\mathbf{fi}} = \frac{\langle \mathrm{g}, \mathbf{k_f}|H_{\mathrm{I}}|e,0\rangle\langle e,0|H_{\mathrm{I}}|\mathrm{g},\mathbf{k_i}\rangle}{\hbar\omega - \hbar\omega_0 + i\pi\sum_{\mathbf{k_f}}|\langle g,\mathbf{k_f}|H_{\mathrm{I}}|e,0\rangle|^2\delta\left(\hbar\omega_{\mathbf{k_f}} - \hbar\omega_0\right)} \tag{1}$$

where $\hbar$ is the reduced Planck constant and $|e,0\rangle$ indicates that the resonance is in its excited state and there is no photon in the medium. Using Fermi's golden rule, we can obtain the optical cross section of the resonance as

$$\sigma(\mathbf{k_i},\omega) = \frac{1}{v_{\mathbf{k_i}}/L^3}\sum_{\mathbf{k_f}}\frac{2\pi}{\hbar}|\mathfrak{I}_{\mathbf{fi}}|^2\delta\left(\hbar\omega_{\mathbf{k_f}} - \hbar\omega_{\mathbf{k_i}}\right) \tag{2}$$

The normalization factor $v_{\mathbf{k_i}}/L^3$ is the power flux of a single incident photon, where $v_{\mathbf{k_i}}$ is the group velocity of the incident photon and L^3 is the quantization volume.

We focus on the resonant cross section at the resonant frequency $\omega_{\mathbf{k}_{\mathrm{f}}} = \omega_{\mathbf{k}_{\mathrm{i}}} = \omega_0$. The summation in the momentum space then can be converted to an integration over the iso-surface S defined by $\omega(\mathbf{k}) = \omega_0$ as

$$\sum_{\mathbf{k_f}}\delta\left(\hbar\omega_{\mathbf{k_f}} - \hbar\omega_0\right) = \left(\frac{L}{2\pi}\right)^3\iint_S\frac{ds}{\hbar v_{\mathbf{k_f}}} \tag{3}$$

The resonant cross section at $\omega_{\mathbf{k}_{\mathrm{f}}} = \omega_{\mathbf{k}_{\mathrm{i}}} = \omega_0$ then can be further calculated as

$$\sigma(\mathbf{k_i},\omega_0) = \frac{2\pi}{\hbar}\frac{|\langle e,0|H_{\mathrm{I}}|g,\mathbf{k}_{\mathrm{i}}\rangle|^2}{v_{\mathbf{k_i}}/L^3}\frac{1}{\pi^2\left(\frac{L}{2\pi}\right)^3\iint_S\frac{ds}{\hbar v_{\mathbf{k_f}}}|\langle g,\mathbf{k_f}|H_{\mathrm{I}}|e,0\rangle|^2}$$

$$= 16\pi^2 \frac{\frac{|\langle e,0|H_\mathrm{I}|g,\mathbf{k}_\mathrm{i}\rangle|^2}{\hbar v_{\mathbf{k_i}}}}{\iint_S \frac{ds}{\hbar v_{\mathbf{k_f}}} |\langle g,\mathbf{k_f}|H_\mathrm{I}|e,0\rangle|^2} \tag{4}$$

Next, we integrate the resonant cross section $\sigma(\mathbf{k_i},\omega_0)$ over the iso-surface S and immediately obtain the conservation law

$$\iint_S \sigma(\mathbf{k_i},\omega_0)ds = 16\pi^2 \frac{\iint_S \frac{ds}{\hbar v_{\mathbf{k_i}}} |\langle e,0|H_I|g,\mathbf{k}_\mathrm{i}\rangle|^2}{\iint_S \frac{ds}{\hbar v_{\mathbf{k}_f}} |\langle g,\mathbf{k_f}|H_\mathrm{I}|e,0\rangle|^2}$$

$$= 16\pi^2 \tag{5}$$

Supplementary Note 2: Quantum scattering theory

The resonant scattering cross section of a single two-level system (TLS) can be calculated using many approaches[2,3]. Here in our work, we calculated the scattering cross section and further numerically verified the conservation law by using the quantum scattering theory we recently developed[4]. In this section, we will describe our quantum scattering theory in detail.

As we described in the main text, the general Hamiltonian governing the interaction between photons and a single TLS is given by[5]

$$H = \hbar\omega_0 b^\dagger b + \sum_{\mathbf{k}} \hbar\omega_{\mathbf{k}} c_{\mathbf{k}}^\dagger c_{\mathbf{k}} + i\hbar \sum_{\mathbf{k}} g_{\mathbf{k}} \left(c_{\mathbf{k}}^\dagger b - c_{\mathbf{k}} b^\dagger\right) \tag{6}$$

Here $b^\dagger$ (b) is the raising (lowering) atomic operator of the TLS. The transition frequency of the TLS is ω_0. The creation (annihilation) operator for photons with angular frequency $\omega_{\mathbf{k}}$ is $c_{\mathbf{k}}^\dagger(c_{\mathbf{k}})$, with $\mathbf{k}$ being the momentum. The coupling strength between the TLS and the photon with momentum $\mathbf{k}$ is given by $g_{\mathbf{k}} = \mathbf{d}\cdot\hat{\mathbf{E}}_{\mathbf{k}}\sqrt{\omega_0/2\hbar\epsilon_0 L^3}$, where $\mathbf{d}$ is the dipole moment of the TLS, $\hat{\mathbf{E}}_{\mathbf{k}}$ is the unit polarization vector of the photons at the location of the TLS and ϵ_0 is the vacuum permittivity.

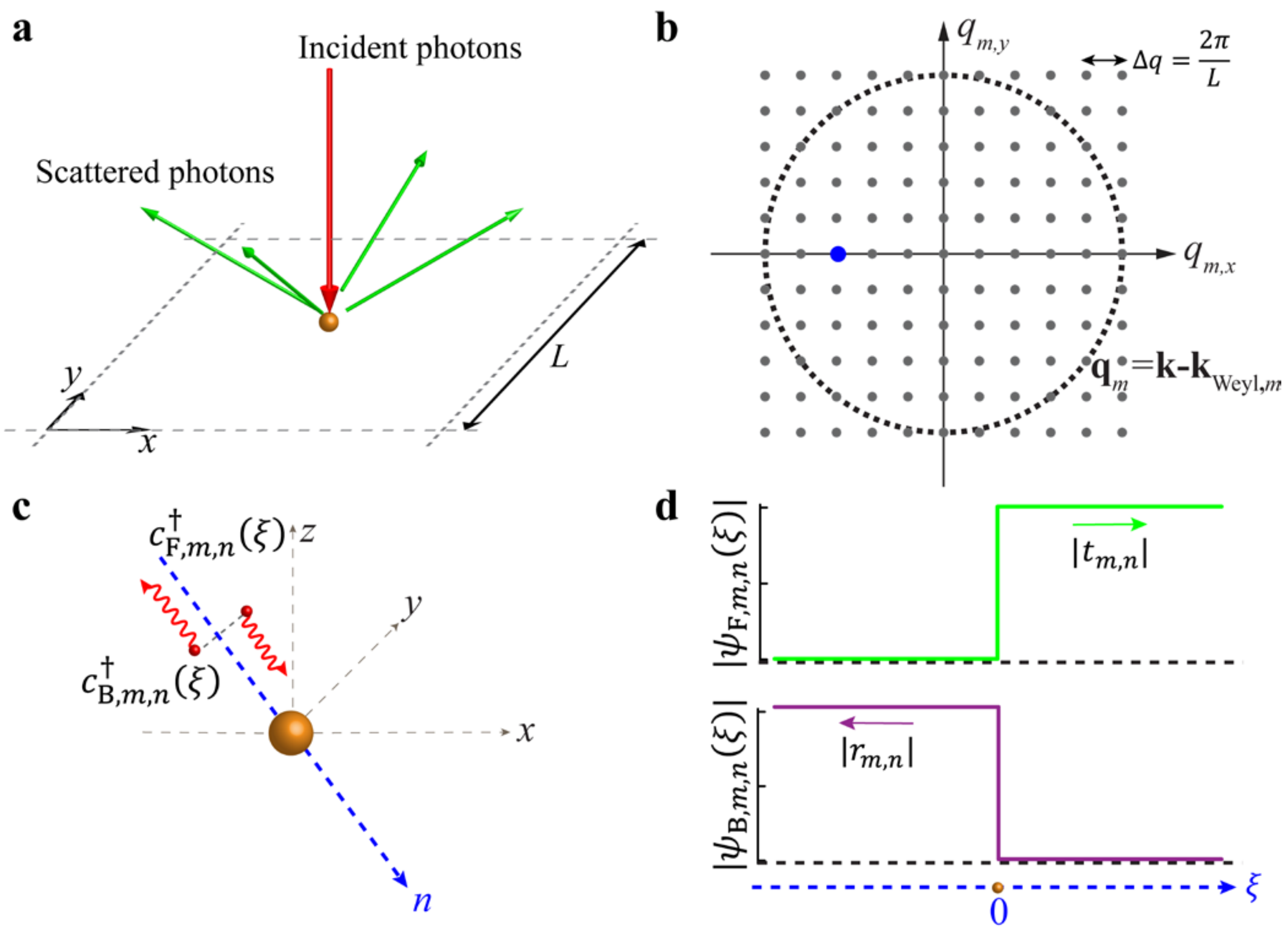

Supplementary Figure 1: Quantum scattering theory. a Schematic of a two-level system in a continuum. Periodic boundary conditions with periodicity L are applied in both x and y directions. Because of the periodicity, incident photons can only be scattered to a set of discretized directions. **b** The $\mathbf{q}_{m,xy}$ space is discretized by $\Delta q = 2\pi/L$, and represents a set of eigenmode channels (grey dots). **c** Real space bosonic creation operators. The real-space operators $c^\dagger_{\mathrm{F},m,n}(\xi)$ and $c^\dagger_{\mathrm{B},m,n}(\xi)$ create a forward and backward propagating single photon at position ξ in the nth channel of the mth Weyl point, respectively. **d** Spatial single-photon wavefunctions $\psi_{\mathrm{F},m,n}(\xi)$ and $\psi_{\mathrm{B},m,n}(\xi)$ in the nth channel of the mth Weyl point. The coefficients $r_{m,n}$ and $t_{m,n}$ are the amplitudes of reflected and transmitted photons in the nth channel of the mth Weyl point, respectively.

The second term $\sum_{\mathbf{k}} \hbar\omega_{\mathbf{k}} c^\dagger_{\mathbf{k}} c_{\mathbf{k}}$ describes the propagating photons in the photonic crystals. Here we focus on the Weyl-point photonic crystal. As we described in the main text, the dispersion is governed by the Weyl Hamiltonian $\mathcal{H}(\mathbf{q}) = v_x q_x \sigma_x + v_y q_y \sigma_y + v_z q_z \sigma_z$, where $\sigma_{x,y,z}$ are Pauli matrices. The momentum $\mathbf{q}(q_x, q_y, q_z) = \mathbf{k} - \mathbf{k}_{\mathrm{Weyl}}$ defines the distance to the Weyl point in the momentum space. Diagonalization of the Weyl Hamiltonian yields a three-dimensional (3D) conical dispersion around the frequency of the Weyl point ω_{Weyl}. To simplify the discussion, we consider isotropic Weyl points where $v_x = v_y = v_z = v$. The dispersion around the Weyl point then can be simplified to $\widetilde{\omega}_{\mathbf{q}} = \omega_{\mathbf{k}} - \omega_{\mathrm{Weyl}} = \pm v|\mathbf{q}|$. Note Weyl points typically appear in pairs and the minimum number of Weyl points is two. Here we assume the number of Weyl points is M and the position of mth Weyl point in the momentum space is

$\mathbf{k}_{\mathrm{Weyl},m}$. By defining $\mathbf{q}_m = \mathbf{k} - \mathbf{k}_{\mathrm{Weyl},m}$, the Hamiltonian in Supplementary Equation (1) can be rewritten as

$$H = \hbar\omega_0 b^\dagger b + \sum_{m=1}^{M}\sum_{\mathbf{q}_m} \hbar\widetilde{\omega}_{\mathbf{q}_m} c_{\mathbf{q}_m}^\dagger c_{\mathbf{q}_m} + i\hbar \sum_{m=1}^{M}\sum_{\mathbf{q}_m} g_{\mathbf{q}_m}\left(c_{\mathbf{q}_m}^\dagger b - c_{\mathbf{q}_m} b^\dagger\right) \tag{7}$$

The key idea of our quantum scattering theory is to convert the summation over $\mathbf{q}_m$ to a summation over a set of 1-dimension (1D) channels, or waveguides, where solutions have been successfully obtained[6]. Each channel carries a eigenmode of the photonic crystal with a distinct momentum $\mathbf{q}_m$. They can be illustrated in the $\mathbf{q}_m$-space as shown in Supplementary Figures 1a-b. Here, we use box quantization for the $\mathbf{q}_m$-space by setting up a periodic boundary condition in both x and y directions. At the end of derivation, we will take the periodicity L to ∞ to effectively remove the impact of this artificial periodic boundary condition. Because of the periodicity, incident photons can only be scattered to a set of discretized channels. These channels are defined by the in-plane wave vectors $\mathbf{q}_{m,xy}$ of the wave, which are discretized by $\Delta q = 2\pi/L$ (Supplementary Figure 1b). For a given frequency $\omega_{\mathbf{q}_m}$, the channels are located on a sphere of radius $|\mathbf{q}_m| = \omega_{\mathbf{q}_m}/v$. Thereby, the total number of channels N is given by $N = \oiint \cos\theta_{\mathbf{q}_m}\, d^2\mathbf{q}_m/(\Delta q)^2\,/2 = \pi(\widetilde{\omega}_{\mathbf{q}_m}/v\Delta q)^2$. We can then convert the summation over $\mathbf{q}_m$ to

$$\sum_{\mathbf{q}_m} \hbar\widetilde{\omega}_{\mathbf{q}_m} c_{\mathbf{q}_m}^\dagger c_{\mathbf{q}_m} = \sum_{n=1}^{N}\sum_{q_{m,n}} \hbar\widetilde{\omega}_{q_{m,n}} c_{q_{m,n}}^\dagger c_{q_{m,n}} \tag{8}$$

$$\sum_{\mathbf{q}_m} g_{\mathbf{q}_m}\left(c_{\mathbf{q}_m}^\dagger b - c_{\mathbf{q}_m} b^\dagger\right) = \sum_{n=1}^{N}\sum_{q_{m,n}} g_{q_{m,n}}\left(c_{q_{m,n}}^\dagger b - c_{q_{m,n}} b^\dagger\right) \tag{9}$$

Note the wave number $q_{m,n}$ is a scalar. It's also useful to differentiate waves with opposite group velocities as their dispersions are governed by two different branches. We define two scalar wave numbers $q_{m,n}^{\mathrm{F}}$ and $q_{m,n}^{\mathrm{B}}$ for forward and backward propagating waves, respectively. The summations over $\mathbf{q}_m$ then become

$$\sum_{\mathbf{q}_m} \hbar\widetilde{\omega}_{\mathbf{q}_m} c_{\mathbf{q}_m}^\dagger c_{\mathbf{q}_m} = \sum_{n=1}^{N}\Big(\sum_{q_{m,n}^{\mathrm{F}}} \hbar\widetilde{\omega}_{q_{m,n}^{\mathrm{F}}} c_{q_{m,n}^{\mathrm{F}}}^\dagger c_{q_{m,n}^{\mathrm{F}}} + \sum_{q_{m,n}^{\mathrm{B}}} \hbar\widetilde{\omega}_{q_{m,n}^{\mathrm{B}}} c_{q_{m,n}^{\mathrm{B}}}^\dagger c_{q_{m,n}^{\mathrm{B}}}\Big) \tag{10}$$

$$\sum_{\mathbf{q}_m} g_{\mathbf{q}_m}\left(c_{\mathbf{q}_m}^\dagger b - c_{\mathbf{q}_m} b^\dagger\right) = \sum_{n=1}^{N}\sum_{q_{m,n}^{\mathrm{F}}} g_{q_{m,n}^{\mathrm{F}}}\left(c_{q_{m,n}^{\mathrm{F}}}^\dagger b - c_{q_{m,n}^{\mathrm{F}}} b^\dagger\right)$$

$$+\sum_{n=1}^{N}\sum_{q_{m,n}^{\mathrm{B}}} g_{q_{m,n}^{\mathrm{B}}}\left(c_{q_{m,n}^{\mathrm{B}}}^\dagger b - c_{q_{m,n}^{\mathrm{B}}} b^\dagger\right) \tag{11}$$

where $\widetilde{\omega}_{q_{m,n}^{\mathrm{F}}} = vq_{m,n}^{\mathrm{F}}$ and $\widetilde{\omega}_{q_{m,n}^{\mathrm{B}}} = -vq_{m,n}^{\mathrm{B}}$ are the dispersion relationships for forward and backward propagating photons in each channel, respectively. Note $q_{m,n}^{\mathrm{F}}$ and $q_{m,n}^{\mathrm{B}}$ are scalars with opposite signs.

Our next step is to convert the $\mathbf{q}_m$-space operators $c_{q_{m,n}^{\mathrm{F}}}^{\dagger}$ and $c_{q_{m,n}^{\mathrm{B}}}^{\dagger}$ to real-space operators. It can be realized by defining the following Fourier transformation

$$c_{q_{m,n}^{\mathrm{F}}}^{\dagger} = \sqrt{\frac{1}{L}}\int_{-L}^{L} d\xi\, c_{\mathrm{F},m,n}^{\dagger}(\xi) e^{iq_m^{\mathrm{F}}\xi} \tag{12}$$

$$c_{q_{m,n}^{\mathrm{B}}}^{\dagger} = \sqrt{\frac{1}{L}}\int_{-L}^{L} d\xi\, c_{\mathrm{B},m,n}^{\dagger}(\xi) e^{iq_m^{\mathrm{B}}\xi} \tag{13}$$

where operators $c_{\mathrm{F},m,n}^{\dagger}(\xi)$ and $c_{\mathrm{B},m,n}^{\dagger}(\xi)$ create a forward and a backward propagating photon at position ξ in the *n^th^* channel, respectively (Supplementary Figure 1c). By using rotating wave approximation, the summations over $q_{m,n}^{\mathrm{F}}$ further become

$$\begin{aligned}
\sum_{q_{m,n}^{\mathrm{F}}} \hbar\widetilde{\omega}_{q_{m,n}^{\mathrm{F}}} c_{q_{m,n}^{\mathrm{F}}}^{\dagger} c_{q_{m,n}^{\mathrm{F}}} &= \sum_{q_{m,n}^{\mathrm{F}}} \frac{\hbar v q_{m,n}^{\mathrm{F}}}{L} \int_{-\infty}^{\infty}\int_{-\infty}^{\infty} d\xi d\xi'\, c_{\mathrm{F},m,n}^{\dagger}(\xi) c_{\mathrm{F},m,n}(\xi') e^{iq_m^{\mathrm{F}}(\xi-\xi')} \\
&= \frac{\hbar v}{\Delta q L}\int_{-\infty}^{\infty}\int_{-\infty}^{\infty} d\xi d\xi'\, c_{\mathrm{F},m,n}^{\dagger}(\xi) c_{\mathrm{F},m,n}(\xi') \int_{-\infty}^{\infty} q_{m,n}^{\mathrm{F}}\, e^{iq_m^{\mathrm{F}}(\xi-\xi')}\, dq_{m,n}^{\mathrm{F}} \\
&= i\hbar v \int_{-\infty}^{\infty}\int_{-\infty}^{\infty} d\xi d\xi'\, c_{\mathrm{F},m,n}^{\dagger}(\xi)(-\frac{d}{d\xi}) c_{\mathrm{F},m,n}(\xi')\delta(\xi-\xi') \\
&= i\hbar v \int_{-\infty}^{\infty} d\xi\, c_{\mathrm{F},m,n}^{\dagger}(\xi)\left(-\frac{d}{d\xi}\right) c_{\mathrm{F},m,n}(\xi)
\end{aligned} \tag{14}$$

and

$$\begin{aligned}
&\sum_{q_{m,n}^{\mathrm{F}}} g_{q_{m,n}^{\mathrm{F}}}\left(c_{q_{m,n}^{\mathrm{F}}}^{\dagger} b - c_{q_{m,n}^{\mathrm{F}}} b^{\dagger}\right) \\
&= \sum_{q_{m,n}^{\mathrm{F}}} g_{q_{m,n}^{\mathrm{F}}} \sqrt{\frac{1}{L}} \left(\int_{-\infty}^{\infty} d\xi\, c_{\mathrm{F},m,n}^{\dagger}(\xi) e^{iq_{m,n}^{\mathrm{F}}\xi} b - \int_{-\infty}^{\infty} d\xi\, c_{\mathrm{F},m,n}(\xi) e^{-iq_{m,n}^{\mathrm{F}}\xi} b^{\dagger}\right) \\
&= \frac{g_{q_{m,n}^{\mathrm{F}}}}{\Delta q}\sqrt{\frac{1}{L}}\left(\int_{-\infty}^{\infty} d\xi\, c_{\mathrm{F},m,n}^{\dagger}(\xi) b \int_{-\infty}^{\infty} dq_{m,n}^{\mathrm{F}} e^{iq_{m,n}^{\mathrm{F}}\xi} - \int_{-\infty}^{\infty} d\xi\, c_{\mathrm{F},m,n}(\xi) b^{\dagger} \int_{-\infty}^{\infty} dq_{m,n}^{\mathrm{F}} e^{-iq_{m,n}^{\mathrm{F}}\xi}\right) \\
&= g_{m,n}\sqrt{L}\int_{-\infty}^{\infty} d\xi\, \delta(\xi)\left(c_{\mathrm{F},m,n}^{\dagger}(\xi) b - c_{\mathrm{F},m,n}(\xi) b^{\dagger}\right)
\end{aligned} \tag{15}$$

Similarly, the summations over q_m^{B} further become

$$\sum_{q_{m,n}^{\mathrm{B}}} \hbar\widetilde{\omega}_{q_{m,n}^{\mathrm{B}}} c_{q_{m,n}^{\mathrm{B}}}^{\dagger} c_{q_{m,n}^{\mathrm{B}}} = i\hbar v \int_{-\infty}^{\infty} d\xi c_{\mathrm{B},m,n}^{\dagger}(\xi)\left(-\frac{d}{d\xi}\right) c_{\mathrm{B},m,n}(\xi) \tag{16}$$

and

$$\sum_{q_{m,n}^{\mathrm{B}}} g_{q_{m,n}^{\mathrm{B}}}\left(c_{q_{m,n}^{\mathrm{B}}}^{\dagger} b - c_{q_{m,n}^{\mathrm{B}}} b^{\dagger}\right) = g_{m,n}\sqrt{L}\int_{-\infty}^{\infty} d\xi \delta(\xi)\left(c_{\mathrm{B},m,n}^{\dagger}(\xi) b - c_{\mathrm{B},m,n}(\xi) b^{\dagger}\right) \tag{17}$$

By substituting Supplementary Equations (8) – (17) into Supplementary Equation (7) we obtain the real-space Hamiltonian as

$$H = \hbar\omega_0 b^{\dagger} b + i\hbar v L \sum_{m=1}^{M}\sum_{n=1}^{N}\int_{-\infty}^{\infty} d\xi\left(c_{\mathrm{F},m,n}^{\dagger}(\xi)\left(-\frac{d}{d\xi}\right) c_{\mathrm{F},m,n} + c_{\mathrm{B},m,n}^{\dagger}(\xi)(\frac{d}{d\xi}) c_{\mathrm{B},m,n}(\xi)\right)$$

$$+ i\hbar \sum_{m=1}^{M}\sum_{n=1}^{N}\int_{-\infty}^{\infty} d\xi g_{m,n} L\delta(\xi)\left\{\left(c_{\mathrm{F},m,n}^{\dagger}(\xi) + c_{\mathrm{B},m,n}^{\dagger}(\xi)\right) b - \left(c_{\mathrm{F},m,n}(\xi) + c_{\mathrm{B},m,n}(\xi)\right) b^{\dagger}\right\} \tag{18}$$

Given the real-space Hamiltonian, our next step is to solve the time-independent Schrodinger equation $H|\psi\rangle = \hbar\omega_{\mathbf{q}}|\psi\rangle$, where $|\psi\rangle$ is the eigenstate. The most general eigenstate can be written in terms of the eigenmodes of the photonic crystal as

$$|\psi\rangle = \sum_{m=1}^{M}\sum_{n=1}^{N}\int d\xi\left[\psi_{\mathrm{F},m,n}(\xi) c_{\mathrm{F},m,n}^{\dagger}(\xi) + \psi_{\mathrm{B},m,n}(\xi) c_{\mathrm{B},m,n}^{\dagger}(\xi)\right]|0,\mathrm{g}\rangle + E b^{\dagger}|0,\mathrm{g}\rangle \tag{19}$$

where $|0,\mathrm{g}\rangle$ indicates that the TLS is in the ground state and E is the excitation amplitude of the TLS. The single-photon wavefunctions $\psi_{\mathrm{F},m,n}(\xi)$ and $\psi_{\mathrm{B},m,n}(\xi)$ in the *n**th* channel of the *m**th* Weyl point are given by

$$\psi_{\mathrm{F},m,n}(\xi) = u(\xi) e^{iq_{m,n}\xi}\left[\delta_{ml}\delta_{np}\theta(-\xi) + t_{m.n}\theta(\xi)\right] \tag{20}$$

$$\psi_{\mathrm{B},m,n}(\xi) = u(\xi) e^{-iq_{m,n}\xi}\left[r_{m,n}\theta(-\xi)\right] \tag{21}$$

where $u(\xi)e^{iq_m\xi}$ is the eigenmode associated with wave number $q_{m,n}$ and $\theta(\xi)$ is the Heaviside step function. The Kronecker delta $\delta_{ml}\delta_{np}$ indicates that single photons are incident from the in the pth channel of the lth Weyl point. The coefficients $r_{m,n}$ and $t_{m,n}$ are the amplitudes of the reflected and transmitted photons in the nth channel of the mth Weyl point, respectively. We schematically plot the wavefunctions in Supplementary Figure 1d for clear visualization.

By substituting the eigenstate into the real-space Hamiltonian, we obtain the following set of equations for each channel

$$-iv\frac{d}{d\xi}\left[\delta_{ml}\delta_{np} u(\xi)\theta(-\xi) + t_{m,n} u(\xi)\theta(\xi)\right] + i g_{m,n}\sqrt{L} e^{-iq_{m,n}\xi} E = 0 \tag{22}$$

$$ive^{iq_{m,n}\xi}\frac{d}{d\xi}r_{m,n}u(\xi)\theta(-\xi)+ig_{m,n}\sqrt{L}e^{iq_{m,n}\xi}E=0 \tag{23}$$

and the following equation for the interaction between the TLS and the propagating waves

$$\omega_0 E-i\sum_{m=1}^{M}\sum_{n=1}^{N}g_{m,n}\sqrt{L}\left(\frac{u(0)\left(\delta_{ml}\delta_{np}+t_{m,n}\right)}{2}+\frac{u(0)r_{m,n}}{2}\right)=\omega_{\mathbf{q}}E \tag{24}$$

By solving Supplementary Equation (22) and Supplementary Equation (23) we can obtain the coefficients t_n and r_n as

$$t_{m,n}=\frac{g_{m,n}\sqrt{L}}{vu(0)}E+\delta_{ml}\delta_{np} \tag{25}$$

$$r_{m,n}=\frac{g_{m,n}\sqrt{L}}{vu(0)}E \tag{26}$$

By substituting Supplementary Equations (25) – (26) into Supplementary Equation (24), we obtain a simple equation of the excitation amplitude E as

$$\left(\omega_{\mathbf{q}}-\omega_0+i\sum_{m=1}^{M}\sum_{n=1}^{N}\frac{\left(g_{m,n}\right)^2L}{v}\right)E=-ig_{l,p}\sqrt{L}u(0) \tag{27}$$

We can then obtain the transmission coefficient $t_{m,n}$ and reflection coefficient $t_{m,n}$ in the *n*th channel of the *m*th Weyl point as

$$t_{m,n}=-i\frac{g_{m,n}g_{l,p}L}{v}\frac{1}{\omega_{\mathbf{q}}-\omega_0+i\frac{\Gamma}{2}}+\delta_{ml}\delta_{np} \tag{28}$$

$$r_{m,n}=-i\frac{g_{m,n}g_{l,p}L}{v}\frac{1}{\omega_{\mathbf{q}}-\omega_0+i\frac{\Gamma}{2}} \tag{29}$$

where $\Gamma=2\sum_{m=1}^{M}\sum_{n=1}^{N}\left(g_{m,n}\right)^2L/v$ is the spontaneous emission rate of the TLS and the factor of 2 results from the forward and backward propagating waves.

Thus, the scattering cross section can be calculated as

$$\sigma\left(\omega_{\mathbf{q}},q_{l,p}\right)=\frac{\sum_{m=1}^{M}\sum_{n=1}^{N}\left(|t_{m,n}-\delta_{ml}\delta_{np}|^2+|r_{m,n}|^2\right)}{1/L^2}$$

$$=\frac{2\left(g_{l,p}\right)^2L^3}{v}\frac{\sum_{m=1}^{M}\sum_{n=1}^{N}\left(g_{m,n}\right)^2L/v}{(\omega_0-\omega_{\mathbf{q}})^2+\frac{\Gamma^2}{4}}$$

$$=\frac{\left(g_{l,p}\right)^2L^3}{v}\frac{\Gamma}{\left(\omega_0-\omega_{\mathbf{q}}\right)^2+\frac{\Gamma^2}{4}} \tag{30}$$

We can further normalize the coupling coefficient $\left(g_{m,n}\right)^2 L/v$ by its maximum $(g_0)^2 L/v$, and define an angular factor $f_{m,n} = \left(g_{m,n}/g_0\right)^2$. Supplementary Equation (30) then can be rewritten to an expression similar to the well-known Breit-Wigner formula as

$$\sigma(\omega_{\mathbf{q}}, q_{l,p}) = \frac{2 f_{l,p} L^2}{\sum_{m=1}^{M} \sum_{n=1}^{N} f_{m,n}} \frac{\frac{\Gamma^2}{4}}{\left(\omega_0 - \omega_{\boldsymbol{q}}\right)^2 + \frac{\Gamma^2}{4}}$$

$$= \frac{4 f_{l,p} L^2 (\Delta q)^2}{|q_{m.n}|^2 \sum_{m=1}^{M} \oint \cos\theta_{m.n} f_{m,n} d\Omega_{m,n}} \frac{\frac{\Gamma^2}{4}}{\left(\omega_0 - \omega_{\mathbf{q}}\right)^2 + \frac{\Gamma^2}{4}}$$

$$= \frac{f_{l,p}}{\mathcal{F}} \frac{16\pi^2 v^2}{(\omega_0 - \omega_{Weyl})^2} \frac{\frac{\Gamma^2}{4}}{\left(\omega_0 - \omega_{\mathbf{q}}\right)^2 + \frac{\Gamma^2}{4}} \qquad (31)$$

where $\Omega_{m,n}$ is the solid angle associated with $q_{m,n}$, $\mathcal{F} = \sum_{m=1}^{M} \oint \cos\theta_{m.n} f_{m,n} d\Omega_{m,n}$ and $f_{l,p}/\mathcal{F}$ indicates the angular distribution of the scattering cross section. The prefactor $16v^2/(\omega_0 - \omega_{\mathrm{Weyl}})^2$ ultimately determines the maximum cross section, which agrees with the conclusion in the main text. Note we have assumed that the isosurface around each Weyl point is isotropic throughout the derivation, *i.e.* $|q_{m.n}| = \left|\omega_{q_{m.n}} - \omega_{\mathrm{Weyl}}\right|/v$. For anisotropic Weyl point, the scattering cross section has the same form and only differs from the isotropic case by a constant.

Supplementary Note 3: Frequency dependence of the spectrum of the average cross section

In the main text, we focus on the maximum of the average cross section when $\omega_{\mathbf{q}} = \omega_0$ and briefly discuss the spectrum of the average cross section in Fig. 4. Here we will discuss more details about the frequency dependence of the spectrum of the average cross section.

The average cross section can be directly calculated by using Supplementary Equation (30). To simplify the discussion, here we will also assume that the isosurface around each Weyl point is identical and isotropic, *i.e.*, $|q_{m.n}| = \left|\omega_{q_{m.n}} - \omega_{\mathrm{Weyl}}\right|/v$. Recall that $(g_0)^2 = d^2\omega_0/2\hbar\epsilon_0 L^3$ and d is the dipole moment of the TLS. Thereby, the spontaneous emission rate Γ can also be calculated as

$$\Gamma = 2\sum_{m=1}^{M}\sum_{n=1}^{N} \frac{\left(g_{m,n}\right)^2 L}{v} = \frac{(g_0)^2 L^3 \left(\omega_0 - \omega_{\mathrm{Weyl}}\right)^2}{4\pi^2 v^3} \sum_{m=1}^{M} \oint \cos\theta_{m.n} f_{m,n} d\Omega_{m,n}$$

$$\frac{d^2\omega_0\left(\omega_0 - \omega_{\mathrm{Weyl}}\right)^2}{8\hbar\epsilon_0\pi^2 v^3} \sum_{m=1}^{M} \oint \cos\theta_{m.n} f_{m,n} d\Omega_{m,n}$$

$$= \wp\left(\omega_0 - \omega_{\mathrm{Weyl}}\right)^2 \tag{32}$$

where $\Omega_{m,n}$ is the solid angle associated with wave number $q_{m,n}$. For simplification, we define a coefficient $\wp = d^2\omega_0 \sum_{m=1}^{M} \oint \cos\theta_{m.n} f_{m,n} d\Omega_{m,n} / 8\hbar\epsilon_0\pi^2 v^3$. Around the Weyl point, the coefficient $\wp$ does not vary significantly with the frequency. As a result, the spontaneous emission rate Γ scales as $\left(\omega_0 - \omega_{\mathrm{Weyl}}\right)^2$.

Next, we calculate the average cross section by integrating the cross section on the isosurfaces and then dividing the integration by the area of the isosurfaces. The spectrum of the average cross section then is given by

$$\bar{\sigma}\left(\omega_{\mathbf{q}}\right) = \frac{(g_0)^2 L^3}{v} \frac{\sum_{m=1}^{M} \oint \cos\theta_{m.n} f_{m,n} d\Omega_{m,n}}{\sum_{m=1}^{M} \oint d\Omega_{m,n}} \frac{\Gamma}{\left(\omega_0 - \omega_{\mathbf{q}}\right)^2 + \frac{\Gamma^2}{4}}$$

$$= \frac{d^2\omega_0}{8M\hbar\epsilon_0\pi v} \sum_{m=1}^{M} \oint \cos\theta_{m.n} f_{m,n} d\Omega_{m,n} \frac{\Gamma}{\left(\omega_0 - \omega_{\mathbf{q}}\right)^2 + \frac{\Gamma^2}{4}}$$

$$= \frac{\pi\wp^2 v^2}{M} \frac{\left(\omega_0 - \omega_{\mathrm{Weyl}}\right)^2}{\left(\omega_0 - \omega_{\mathbf{q}}\right)^2 + \frac{\wp^2\left(\omega_0 - \omega_{\mathrm{Weyl}}\right)^4}{4}} \tag{33}$$

Note the prefactor $\pi\wp^2 v^2/M$ does not vary significantly with the frequency around the Weyl point. On resonance, *i.e.*, $\omega_{\mathbf{q}} = \omega_0$, the resonant average cross section scales as $1/\left(\omega_0 - \omega_{\mathrm{Weyl}}\right)^2$ and increases drastically as $\omega_0 \to \omega_{\mathrm{Weyl}}$. In great contrast, the cross section of off-resonance frequencies scales as $\left(\omega_0 - \omega_{\mathrm{Weyl}}\right)^2/\left(\omega_0 - \omega_{\mathbf{q}}\right)^2$ and is strongly suppressed around the Weyl point.

Meanwhile, it's clear to see that the integration of the average cross section $\int_{-\infty}^{\infty} \bar{\sigma}\left(\omega_{\mathbf{q}}\right) d\omega_{\mathbf{q}}$ is roughly a constant, which can be shown as

$$\int_{-\infty}^{\infty} \bar{\sigma}\left(\omega_{\boldsymbol{q}}\right) d\omega_{\boldsymbol{q}} = \int_{-\infty}^{\infty} d\omega_{\boldsymbol{q}} \frac{\pi\wp^2 v^2}{M} \frac{\left(\omega_0 - \omega_{\mathrm{Weyl}}\right)^2}{\left(\omega_{\boldsymbol{q}} - \omega_0\right)^2 + \frac{\wp^2\left(\omega_0 - \omega_{\mathrm{Weyl}}\right)^4}{4}}$$

$$= \frac{2\wp\pi^2 v^2}{M} \tag{34}$$

where $2\wp\pi^2 v^2/M$ remains around the same around the Weyl point.

Supplementary Note 4: Resonant scattering in Dirac systems

The conservation law of resonant scattering can be also extended to two-dimensional (2D) space. In 2D space, the conical dispersion forms a Dirac point, which is the 2D analogy of the Weyl point. In this section, we will use a 2D Dirac photonic crystal as an example and demonstrate the diverging resonant scattering cross section around the Dirac point.

We consider a triangular lattice of dielectric rods (grey circle in Supplementary Figure 2a), which has a dielectric constant of $\epsilon = 12$. The radius of the rods is $0.3a$, where a is the lattice constant. We then solve the band structure of this Dirac photonic crystal by using MPB[7]. We obtain 6 Dirac points in the band structure for the transverse electric (TE) bands at $\omega = 0.462\ (2\pi c/a)$. To visualize the momentum space in 2D space, we plot the isofrequency contour for $\omega = 0.461\ (2\pi c/a)$ in Supplementary Figure 2b. For each Dirac point, the isofrequency contour is almost a circle.

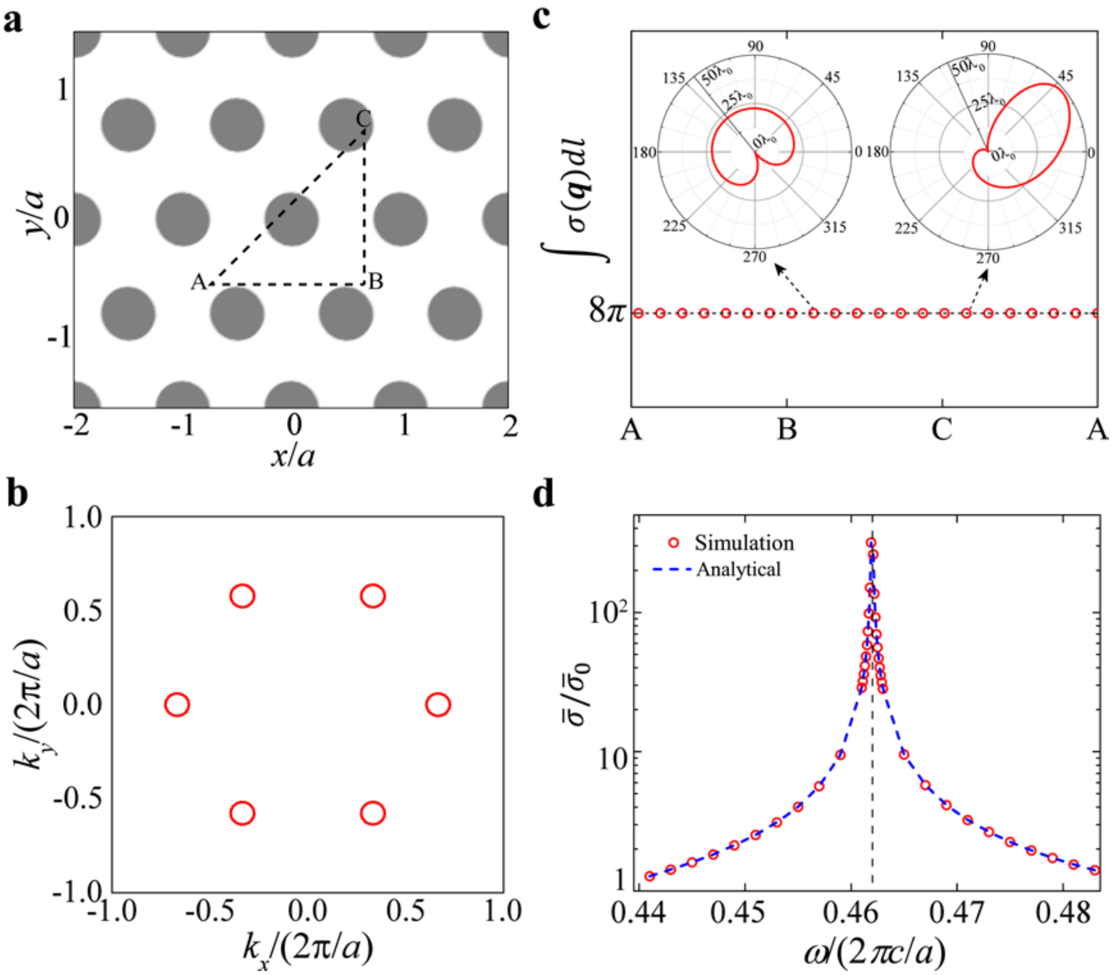

Supplementary Figure 2: Resonant scattering in Dirac systems. a Schematic of the 2D Dirac photonic crystal. **b** Isofrequency contour of the Dirac photonic crystal at $\omega = 0.461\ (2\pi c/a)$. 6 Dirac points are obtained at $\omega = 0.462\ (2\pi c/a)$ for the TE bands. The isofrequency contour is almost a circle. **c** The resonant cross section of the TLS for different locations. The resonant frequency of the TLS is $\omega_0 = 0.461\ (2\pi c/a)$. The integration in momentum space always leads to the same constant. Positions A-B-C-A are labelled in **a**. Examples of $\sigma(\mathbf{q})$ at two different positions are plotted as insets. **d** Diverging

average resonant cross section is realized around the Dirac frequency. The results from quantum scattering simulation (red circles) agree well with the prediction based on the band structure (blue dashed line). The cross section is normalized by the average cross section in free space $\bar{\sigma}_0 = 2\lambda/\pi$. The black dashed line indicates the Dirac frequency.

Next, we numerically verify the conservation law of resonant scattering in Dirac systems. As an example, we consider a TLS with a transition frequency of $\omega_0 = 0.461\ (2\pi c/a)$. The dipole moment of the TLS is in the x direction. The calculated cross sections $\sigma(\mathbf{q})$ of the TLS at two different positions are plotted as insets of Supplementary Figure 2c. Similar to the cross section in Weyl photonic crystals, it also strongly depends on the incident wavevector $\mathbf{q} = \mathbf{k} - \mathbf{k}_{\mathrm{Dirac}}$ and varies significantly at different locations.

The conversation law of resonant cross section in 2D space is slightly different from that in 3D space. Instead of integrating over the isosurface, we now integrate the resonant cross section over the isofrequency contour and the conservation law becomes

$$\int \sigma(\mathbf{q})dl = 8\pi \tag{35}$$

To confirm the above conservation law, we integrate the resonant cross section for a TLS at 16 different locations and plot the results in Supplementary Figure 2c. As expected, the integration $\int \sigma(\mathbf{q})dl$ always results in the same constant of 8π.

We then sweep the transition frequency of the TLS to across the Dirac point, which is indicated by black dashed line in Supplementary Figure 2d. To better demonstrate the enhancement, we also define an average cross section $\bar{\sigma} = \int \sigma(\mathbf{q})dl\,/C$, where C is the total circumference of the isofrequency contours. The calculated average cross section is plotted as red circles in Supplementary Figure 2d. It's enhanced by almost three orders of magnitude compared to $\bar{\sigma}_0 = 2\lambda/\pi$, the average cross section in free space. At the Dirac frequency, the resonant cross section diverges.

Supplementary Note 5. Proof of suppressed Rayleigh (non-resonant) scattering in Weyl systems

The suppressed Rayleigh scattering in Weyl system can be directly proved by using perturbation theory and Born approximation. In this section, we will describe the mathematical proof in detail.

Under the framework of the first-order Born approximation[8], the scattering amplitude $f(\mathbf{k}_s, \mathbf{k}_{\mathrm{inc}})$for a Rayleigh scatterer with a weak scattering potential $\mathbf{V}(\mathbf{r})$ is given by

$$f(\mathbf{k}_s, \mathbf{k}_{\mathrm{inc}}) = \int d^3\mathbf{r}'\ \mathbf{u}_{\mathbf{k}_s}\left(\mathbf{r}'\right)\mathbf{V}\left(\mathbf{r}'\right)\mathbf{u}_{\mathbf{k}_{\mathrm{inc}}}\left(\mathbf{r}'\right)e^{i(\mathbf{k}_{\mathrm{inc}}-\mathbf{k}_s)\cdot\mathbf{r}'} \tag{36}$$

Here $\mathbf{u}_{\mathbf{k}}e^{i\mathbf{k}\cdot\mathbf{r}}$ is the eigenmode of the surrounding medium associated with wave vector $\mathbf{k}$. $\mathbf{k}_s$ and $\mathbf{k}_{\mathrm{inc}}$ are the wave vectors of the scattered and incident eigenmodes, respectively. For simplicity, we assume the scatterer is very small compared to the incident wavelength and can be considered as a point scatterer located at $\mathbf{r}_0$. The scattering potential then can be written as $\mathbf{V}(\mathbf{r}) = \omega\mathbf{V}\delta(\mathbf{r} - \mathbf{r}_0)$. Note here $\mathbf{V}$ is a tensor containing the dielectric constant of the scatterer. Thus, the scattering amplitude becomes

$$f(\mathbf{k}_\mathrm{s}, \mathbf{k}_\mathrm{inc}) = \omega \mathbf{u}_{\mathbf{k}_\mathrm{s}}(\mathbf{r}_0)\mathbf{V}\mathbf{u}_{\mathbf{k}_\mathrm{inc}}(\mathbf{r}_0)e^{i(\mathbf{k}_\mathrm{inc}-\mathbf{k}_\mathrm{s})\cdot\mathbf{r}_0} \tag{37}$$

The total scattering cross section then can be calculated as

$$\sigma(\omega, \mathbf{k}_\mathrm{inc}) = \sum_{\mathbf{k}_\mathrm{s}} |f(\mathbf{k}_\mathrm{s}, \mathbf{k}_\mathrm{inc})|^2 = \omega^2 \sum_{\mathbf{k}_\mathrm{s}} \left|\mathbf{u}_{\mathbf{k}_\mathrm{s}}(\mathbf{r}_0)\mathbf{V}\mathbf{u}_{\mathbf{k}_\mathrm{inc}}(\mathbf{r}_0)\right|^2 \tag{38}$$

The summation over $\mathbf{k}_\mathrm{s}$ can be readily converted to a surface integral over the isosurface given by $\omega(\mathbf{k}_\mathrm{s}) = \omega$, and Supplementary Equation (38) further becomes

$$\sigma(\omega, \mathbf{k}_\mathrm{inc}) = \omega^2 \iint_{S:\omega(\mathbf{k}_\mathrm{s})=\omega} d^2\mathbf{k}_\mathrm{s} \left|\mathbf{u}_{\mathbf{k}_\mathrm{s}}(\mathbf{r}_0)\mathbf{V}\mathbf{u}_{\mathbf{k}_\mathrm{inc}}(\mathbf{r}_0)\right|^2 \propto \omega^2 S \tag{39}$$

where S is the area of the isosurface. In free space, the area of the isosurface S scales $S \sim \omega^2$. Consequently, the Rayleigh scattering cross section scales as $\sigma(\omega, \mathbf{k}_\mathrm{inc}) \sim \omega^4$. In great contrast, the area of the isosurface S scales $S \sim \Delta\omega^2 = (\omega - \omega_\mathrm{Weyl})^2$ and thus the Rayleigh scattering cross section scales as $\sigma(\omega, \mathbf{k}_\mathrm{inc}) \sim \omega^2(\omega - \omega_\mathrm{Weyl})^2$ in Weyl systems.

Supplementary Note 6. Numerical calculation of Rayleigh (non-resonant) scattering in Weyl systems

The Rayleigh scattering cross section can be rigorously calculated by using the normal-mode expansion of the dipole field[9–11] that radiated by the scatterer. In this section, we will briefly describe the normal-mode expansion method that we implemented to numerically calculate the Rayleigh scattering cross section.

In a nonmagnetic medium with a dielectric constant of $\epsilon(\mathbf{r})$, the Maxwell's equations are given by

$$\nabla \times \mathbf{E} = -\mu_0 \frac{\partial \mathbf{H}}{\partial t} \tag{40a}$$

$$\nabla \times \mathbf{H} = -\epsilon(\mathbf{r})\epsilon_0 \frac{\partial \mathbf{E}}{\partial t} + \mathbf{J} \tag{40b}$$

$$\nabla \cdot (\epsilon(\mathbf{r})\epsilon_0 \mathbf{E}) = 0 \tag{40c}$$

$$\nabla \cdot \mathbf{H} = 0 \tag{40d}$$

where μ_0 is vacuum permeability. The electric (magnetic) field is denoted by $\mathbf{E}$ ($\mathbf{H}$). An electromagnetic field is produced by a current density $\mathbf{J}$. Since the charge density is zero, the scalar potential is zero. The electric and magnetic fields can be written in terms of vector potential $\mathbf{A}$ as

$$\mathbf{E} = -\frac{\partial \mathbf{A}}{\partial t} \tag{41a}$$

$$\mathbf{H} = \frac{1}{\mu_0} \nabla \times \mathbf{A} \tag{41b}$$

Thus, the wave equation for the vector potential $\mathbf{A}$ is given by

$$\nabla \times \nabla \times \mathbf{A} + \frac{\epsilon(\mathbf{r})}{c^2}\frac{\partial^2 \mathbf{A}}{\partial^2 t} = \mu_0 \mathbf{J} \tag{42}$$

where c is the speed of light.

The homogeneous solutions to the above wave equation can be written as a superposition of the eigenmodes $\mathbf{A}_\mathbf{k}(\mathbf{r}) = \mathbf{u}_\mathbf{k}(\mathbf{r})e^{i\mathbf{k}\cdot\mathbf{r}}$ of the photonic crystal, which satisfy the homogenous wave equation

$$\nabla \times \nabla \times \mathbf{A}_\mathbf{k}(\mathbf{r}) + \frac{\omega_\mathbf{k}^2}{c^2}\epsilon(\mathbf{r})\mathbf{A}_\mathbf{k}(\mathbf{r}) = 0 \tag{43}$$

The eigenmodes $\mathbf{A}_\mathbf{k}(\mathbf{r})$ also have to satisfy the orthogonalization, normalization and closure conditions given by

$$\int d^3\mathbf{r}\,\epsilon(\mathbf{r})\mathbf{A}_\mathbf{k}(\mathbf{r})\mathbf{A}^*_{\mathbf{k}'}(\mathbf{r}) = (2\pi)^3 L^3 \delta(\mathbf{k} - \mathbf{k}') \tag{44}$$

Here L^3 is the normalization volume.

We then consider a generic Rayleigh scatterer embedded in such a medium. During the Rayleigh scattering process, the incident wave induces an oscillating dipole moment $\mathbf{d} = \alpha\mathbf{E}_{\text{inc}}(\mathbf{r}_0)$ in the scatterer. Here α is the polarizability of the scatterer, and $\mathbf{E}_{\text{inc}}(\mathbf{r}_0)$ is the local incident electric field at the position of the scatterer. Since the scatterer is quite small compared to the wavelength, the external current source $\mathbf{J}$ can be written as

$$\mathbf{J}(\mathbf{r}, t) = -i\omega_0 \mathbf{d}\delta(\mathbf{r} - \mathbf{r}_0)e^{-i\omega_0 t} \tag{45}$$

where $\mathbf{r}_0$ is the position of the scatterer. The vector potential $\mathbf{A}(\mathbf{r}, \mathrm{t})$ then can be obtained by solving Supplementary Equation (42) in terms of $\mathbf{J}$ via the dyadic Green's function $\mathbf{G}$ as

$$\mathbf{A}(\mathbf{r}, t) = \int_{-\infty}^{\infty} dt' \int d^3\mathbf{r}' \; \mathbf{J}\left(\mathbf{r}', t\right)\mathbf{G}\left(\mathbf{r}, t; \mathbf{r}', t'\right) \tag{46}$$

The exact expression of the dyadic Green's function $\mathbf{G}$ can be found in Supplementary Reference [8] and we will not discuss the technical details here. Given the vector potential $\mathbf{A}(\mathbf{r}, t)$, one can show that the time-average radiation power from the induced dipole moment $\mathbf{d}$ is given by[11]

$$P(\omega) = \frac{\pi\omega^2}{4\epsilon_0} \iint_{\omega(\mathbf{k})=\omega} d^2\mathbf{k} \frac{|\mathbf{u}_\mathbf{k}(\mathbf{r}_0) \cdot \mathbf{d}|^2}{v_{g,\mathbf{k}}} \tag{47}$$

Here $v_{g,\mathbf{k}}$ is the group velocity associated with wave vector $\mathbf{k}$. Then the Rayleigh scattering cross section can be calculated as

$$\sigma(\omega, \mathbf{k}_{\text{inc}}) = \frac{P(\omega)}{\frac{1}{2\mu_0} \mathbf{u}_{\mathbf{k}_{\text{inc}}}(\mathbf{r}_0) \times \left(\nabla \times \mathbf{u}^*_{\mathbf{k}_{\text{inc}}}(\mathbf{r}_0)\right)} \tag{48}$$

Here we assume the incident wave is one of the eigenmodes associated with wave vector $\mathbf{k}_{\text{inc}}$. The denominator in Supplementary Equation (48) indicates the Poynting vector of the incident wave at the location of the scatterer, which usually does not vary much with the frequency. On the other hand, Supplementary Equation (47) clearly indicates that the radiation power $P(\omega)$ is proportional to the area of the isosurface S as $P(\omega) \sim \omega^2 S$. Consequently, the Rayleigh scattering cross scales as $\sigma \sim \omega^2 S$, which is consistent with Supplementary Equation (39) that we derived by using perturbation theory.

Supplementary References

1. Zhu, S.-Y., Yang, Y., Chen, H., Zheng, H. & Zubairy, M. S. Spontaneous Radiation and Lamb Shift in Three-Dimensional Photonic Crystals. *Phys. Rev. Lett.* **84,** 2136–2139 (2000).
2. Cohen-Tannoudji, C., Dupont-Roc, J., Grynberg, G. & Thickstun, P. *Atom-photon interactions: basic processes and applications*. (Wiley Online Library, 1992).
3. Dung, H. T., Knöll, L. & Welsch, D.-G. Resonant dipole-dipole interaction in the presence of dispersing and absorbing surroundings. *Phys. Rev. A* **66,** 063810 (2002).
4. Liu, J., Zhou, M. & Yu, Z. Quantum scattering theory of a single-photon Fock state in three-dimensional spaces. *Opt Lett* **41,** 4166–4169 (2016).
5. Ficek, Z. & Swain, S. *Quantum Interference and Coherence: Theory and Experiments*. (Springer Science & Business Media, 2005).
6. Shen, J.-T. & Fan, S. Coherent Single Photon Transport in a One-Dimensional Waveguide Coupled with Superconducting Quantum Bits. *Phys. Rev. Lett.* **95,** 213001 (2005).
7. Johnson, S. G. & Joannopoulos, J. D. Block-iterative frequency-domain methods for Maxwell's equations in a planewave basis. *Opt. Express* **8,** 173–190 (2001).

8. Sakurai, J. J. & Napolitano, J. *Modern Quantum Mechanics*. (Addison-Wesley, 2011).
9. Dowling, J. P. & Bowden, C. M. Atomic emission rates in inhomogeneous media with applications to photonic band structures. *Phys. Rev. A* **46,** 612–622 (1992).
10. Sakoda, K. & Ohtaka, K. Optical response of three-dimensional photonic lattices: Solutions of inhomogeneous Maxwell's equations and their applications. *Phys. Rev. B* **54,** 5732–5741 (1996).
11. Chigrin, D. N. Radiation pattern of a classical dipole in a photonic crystal: Photon focusing. *Phys. Rev. E* **70,** 056611 (2004).
